# Supplementary figures and images for: Extant interspecific hybridization among trematodes within the Schistosoma haematobium species complex in Nigeria
Source: PLoS Negl Trop Dis. 2024 Apr 15;18(4):e0011472. doi: 10.1371/journal.pntd.0011472 (PMC11045100; doi:10.1371/journal.pntd.0011472)

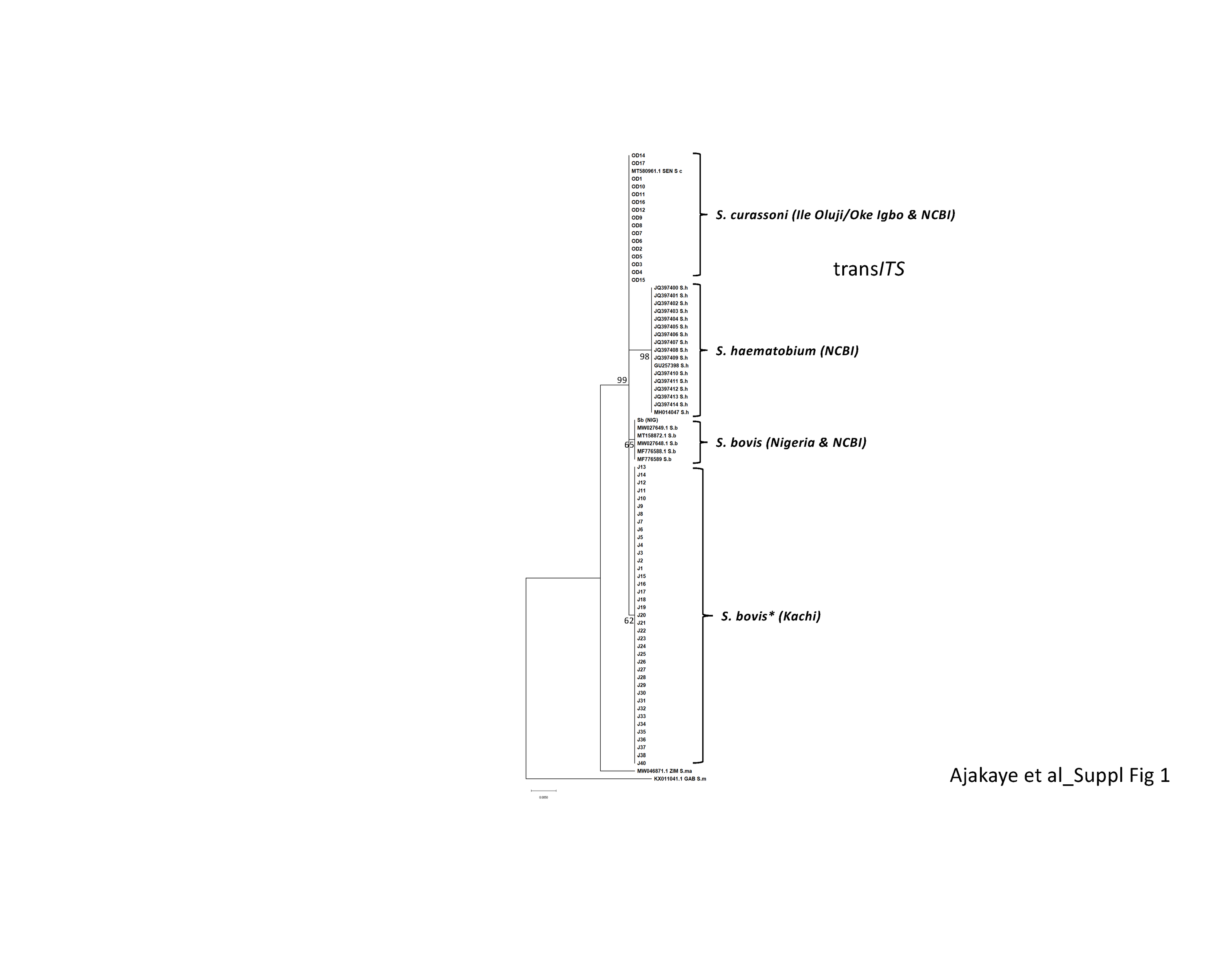

Supplement: S1 Fig — For samples OD14 and OD17, only an S. curassoni allele was recovered at transITS. All other samples were heterozygous and possessed an S. haematobium allele x S. curassoni allele. Only the S. curassoni allele from OD is depicted for comparison against reference alleles for the following: S. bovis Nig = S. bovis worm recovered from a cow in Nigeria. S. bovis (NCBI) = MW027649.1, MT158872.1, MW027648.1, MF776588.1, MF776589.1. S. haematobium (NCBI) = MH014047, GU257398, JQ397400, JQ397401, JQ397402, JQ397403, JQ397404, JQ397405, JQ397406, JQ397407, JQ397408, JQ397409, JQ397410, JQ397411, JQ397412, JQ397413, JQ397414. S.c = S. curassoni isolate from SEN (Senegal), GenBank Accession number MT580961. S. ma = S. mattheei isolate from ZIM (Zimbabwe), GenBank Accession number MW04687. S. m = S. mansoni isolate from GAB (Gabon), GenBank Accession number KX011041. (TIF) [file pntd.0011472.s001.tif]

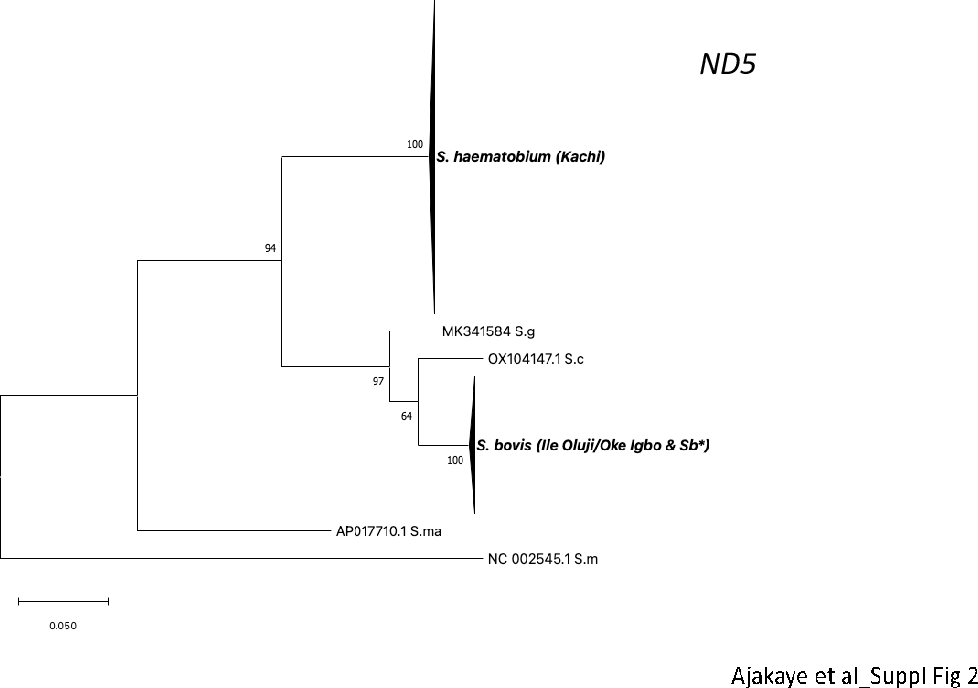

Supplement: S2 Fig — Phylogenetic analysis of ND5 alleles recovered from S. haematobium samples in Oluji/Oke Igbo (OD) and Kachi, (J) in Nigeria. Only S. bovis alleles were identified among samples recovered from Oluji/Oke Igbo (OD), whereas only S. haematobium alleles were identified among samples recovered from Kachi, (J). * This study, (S. bovis worm from cow in Nigeria), S.g = S. guineensis, S.c = S. curassoni, S.m = S. mansoni. (TIF) [file pntd.0011472.s002.tif]

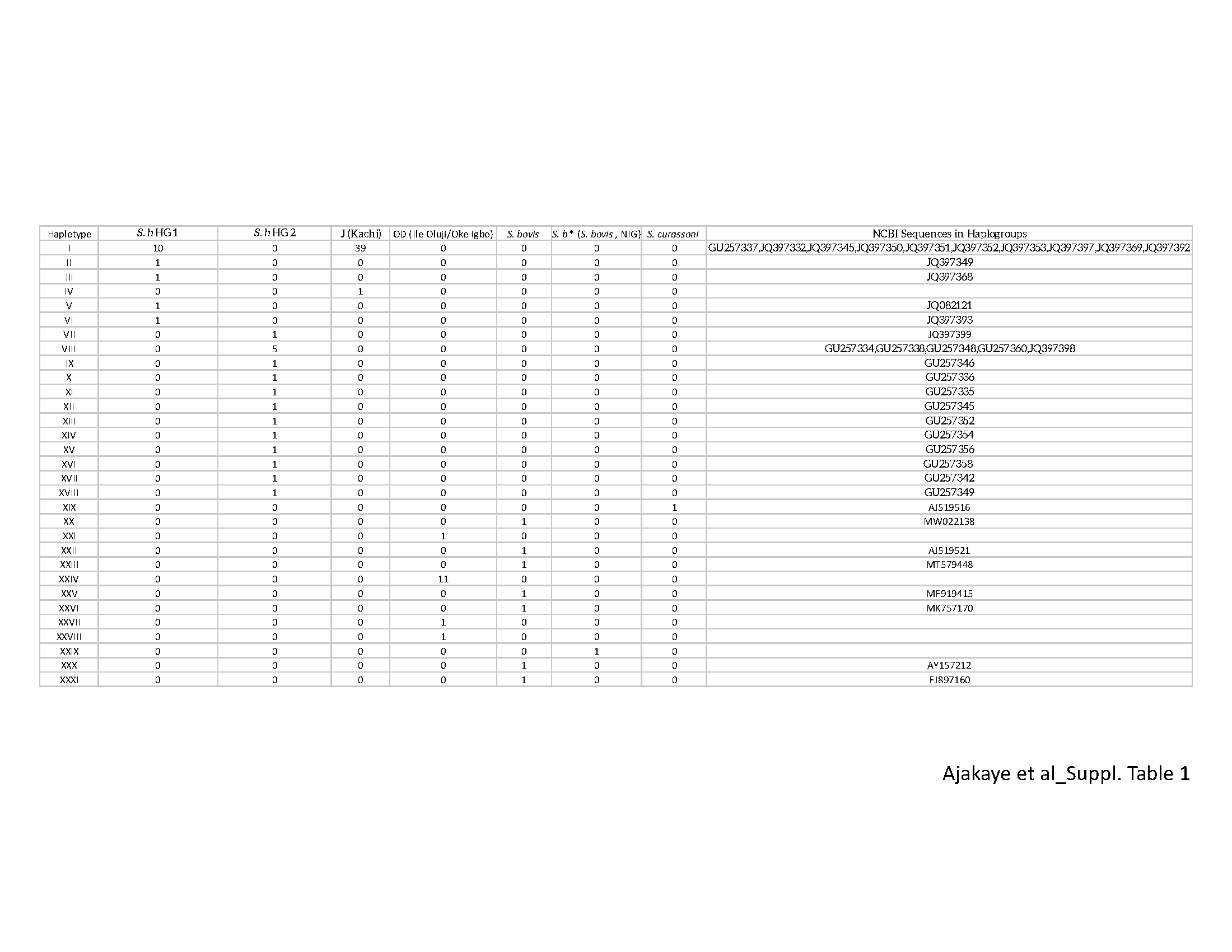

Supplement: S1 Table — (TIFF) [file pntd.0011472.s003.tiff]

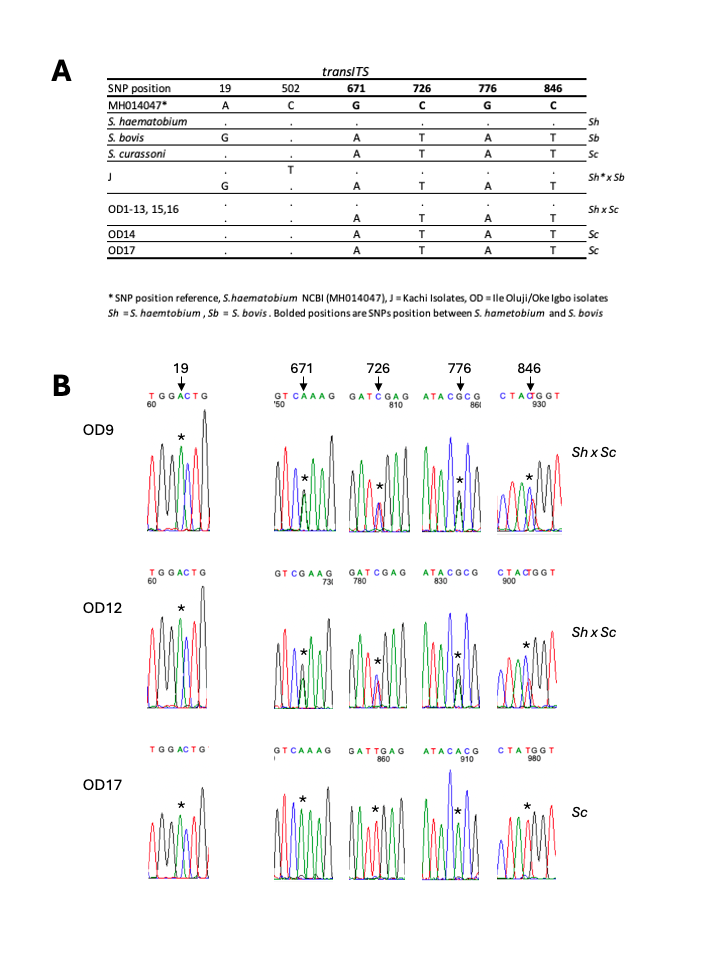

Supplement: S2 Table — A. * SNP position reference, S.haematobium NCBI (MH014047), J = Kachi Samples, OD = Ile Oluji/Oke Igbo samples, Sh = S. haematobium, Sb = S. bovis, Sc = S. curassoni. Each row represents a phased haplotype that was resolved. B. Representative DNA sequence electropherogram profiles at the transITS PCR-amplified population for OD samples that either possess alleles consistent with an Sh x Sc hybrid genotype (OD9, OD12) or an Sc genotype (OD17). Dye peaks denoted by asterisks at positions 671, 726, 776, 846 based on the published sequence (GenBank accession no. MH014047) clearly depict the presence of two nucleotides with similar peak heights, whereas only a single, homozygous “A” nucleotide was resolved at position 19. (TIFF) [file pntd.0011472.s004.tiff]
