# Supplementary material for: Extant interspecific hybridization among trematodes within the Schistosoma haematobium species complex in Nigeria
Source: PLoS Negl Trop Dis. 2024 Apr 15;18(4):e0011472. doi: 10.1371/journal.pntd.0011472 (PMC11045100; doi:10.1371/journal.pntd.0011472)
Supplement: Attachment — Submitted filename: 23 PNTD-D-23-00787_Response_103023A.docx [file pntd.0011472.s005.docx]

"Extant interspecific hybridization among trematodes within the *Schistosoma haematobium* species complex in Nigeria"

*We thank the reviewers for their positive comments, we have revised our manuscript accordingly and trust that it is now acceptable for publication. Specifically, we have added more details to our methods section, including the primers used and the rationale for the markers used or developed and how we phased alleles to produce haplotypes for the phylogenetic analyses we performed. In our revised submission, we also clarify that we are working on DNA extracted from eggs filtered from the urine of positive patients, and we have added the requested details regarding our ethics approvals and that participants were informed of their results. We believe our manuscript is much improved. Please find our responses to the reviewer’s comments (in blue), a “clean” and “track-changes” copy of the revised paper, plus our revised figures.*

**Reviewer #1:**

Generally the methods need more detail added.

Line 224 – Ethics statement. There is no detail regarding what happened to the participants if they were found to be schistosomiasis positive.

*We thank the reviewer for bringing this to our attention, we have added more details to the ethics statement. Please see lines 237-244 in our revised submission.*

Line 246-252 – it is not clear how the urines were identified as positive. You say positive urines were filtered for egg collection. From reading the text it is not clear if this was used to identify positive samples. Also, say how much urine was filtered.

*We thank the reviewer for bringing this to our attention, we have provided more details in the manuscript. Specifically, about 20 ml of urine was collected, please see line 241. For analysis, ~10 ml of urine was drawn from the bottom of every specimen bottle, filtered, and examined for eggs by microscopy (to identify positives) prior to being adsorbed onto membrane filters for subsequent molecular analysis. Please see lines 261-269 in our revised submission.*

Line 254-256 – this information is not clear. Firstly, how was the filter/eggs treated before extraction as they were preserved in Ethanol. You say five Schistosoma species were used as positive controls – do you mean DNA from these species? Also, clearly state where the controls came from (source and origin) and use references.

*We thank the reviewer for bringing this to our attention, the section has been revised to address the questions raised by the reviewer. See lines 276-281 in the revised manuscript. Yes, it was extracted DNA from the five* Schistosoma *spp. that were used as positive controls for the molecular and phylogenetic analyses performed in this submission. We have added the source and origin of the material. Please see revised submission, lines 278-281.*

Line 256-257 – was the filter removed from the lysate before using the Qiagen columns? Also, how much water was used for the elutions?

*We thank the reviewer for bringing this to our attention, the questions have been addressed in the section above, please see line 272-275 in the revised manuscript. Yes, the filters were removed after overnight lysis.*

Line 259 – is transITS used in other papers? – it is typically referred to as ITS1+2 – changing the name may cause confusion. Also, when you say “isolates” do you mean samples?

*We thank the reviewer for bringing this to our attention. We prefer to keep using the term trans*ITS *as it is a general term utilized across a wide array of different parasite species, including protists, fungi, and some helminths. It is more precise, as the primers we used are anchored in the 18S and 28S, and amplify across to include the ITS1, 5.8S, and ITS2. We appreciate the reviewer pointing out to us that this may impart some confusion, in our revised manuscript, we added the following … transITS,* *which includes the ITS1, 5.8S, and ITS2 region… please see line 66 in the revised manuscript.*

*We apologize for any confusion regarding our labeling the DNA extracted from pools of ova from the filters as “isolates”. That is incorrect, and we appreciate the reviewer pointing this out to us, and we have carefully gone back through the paper, and replaced all mention of “isolates” with the more precise “samples” designation.*

Line 261-268 – as you are using primers that you have newly developed you should explain how these have been developed and tested.

*We thank the reviewer for bringing this to our attention, the section has been revised and these essential details have been added.*

Line 263 – it is not clear why you are designed a S. bovis specific PCR as you are also using cox1 and ND5 data (pan genus) that is sequenced. You also do not say which molecular target your S. bovis specific PCR is for.

*We thank the reviewer for bringing this to our attention. This was developed for our own initial screens to determine if S. bovis alleles were present. But once we switched to the pan-genus primers, these became moot. We agree, its simpler to remove from the paper, to avoid any confusion.*

Line 265 – the new target “Bravo-Figey domain needs some information. How was this identified and how were the primers designed?

*We thank the reviewer for bringing this to our attention, a similar question was raised by Reviewer #2. The section has been revised and these essential details added, please see lines 288-305 accordingly:*

*“We also developed a new, unlinked nuclear genome marker (BF) to assess incongruence, BF was targeted because it is a surface antigen that is polymorphic between different schistosome species within the S. haematobium species complex, and is under positive selection. In our previous work examining population genetic structures among various protists, surface antigen genes were identified as excellent genetic markers for phylogenetic analyses, largely because they are typically under diversifying pressure, and are sufficiently polymorphic. In this case, the Bravo Figey or NrCAM is a highly conserved protein which is part of the intracellular region of the neural adhesion molecule L1 proteins. The role of cell adhesion molecules in host parasite interactions has been recognized. They have been suggested as mediators of attachment of pathogens to host cells in protozoan parasites infections [27]. In Trichobilharzia, CAMs regulate cell adhesion to maintain their structure. They are upregulated in the schistosomula of T. regenti suggesting rapid growth and development of different organ structures within the definitive host [28]. Importantly the single gene copy BF marker (chromosome 7) is unlinked from transITS (chromosome 2), which allowed us to look for samples that were incongruent (i.e., chromosomes of distinct ancestry), and thus support a meiotic process in the origin of the sample genotyped.”*

Line 268-269 – you say you sequenced DNA from the 5 controls – which targets were sequenced?

*We thank the reviewer for bringing this to our attention. In our revised manuscript, we have indicated that the DNA from all five species were PCR-amplified and Sanger sequenced at the 4 markers used in our study, please refer to lines 276-278.*

Line 270 – you do not described the PCR’s for the cox1 and ITS.

*We thank the reviewer for bringing this to our attention, that information has been added to our revised submission, please see lines 281-285.*

Line 276-284 – you need to add more information to your analyses. E.g. what type of phylogenetic analysis was done? Which molecular targets were analysed by which method. What are your fragment lengths that were analysed. Was the ITS data analysed looking at the species specific SNP regions?

*We thank the reviewer for bringing this to our attention, this section was revised and now describes the alignment and phylogenetic analysis applications used to produce the tree, including the size of the target aligned for each marker. Please see lines 329-335. To answer the specific question raised about the ITS – 880 nucleotides were included, and Supplemental Table 2 (provided in the original version of our submitted manuscript) highlights the species-specific SNPs.*

Results and Discussion

Unfortunately the way the results are written make it very difficult to understand and interpret. More clarity is needed. It would help if the results and discussion were to be separated so the results can clearly be shown without incorporation of the discussion text, which makes it hard to follow.

*We thank the reviewer for their perspective, but we prefer to combine the results and discussion, to make specific discussion points within the context of each result presented.*

Lines 122 and 123 – the use of “aggregate” and “agrarian” is used but this just adds confusion and you do not explain what these terms mean.

*We thank the reviewer for bringing this to our attention, we agree. We have changed “aggregate” to “total” to avoid this confusion. We want “agrarian” to refer specifically to an agricultural region, please see line 165 in our revised submission.*

Table 1 – it is not clear what intensity is based on. Was it 10 mls of filtered urine?

*We thank the reviewer for bringing this to our attention. 10mls of urine was filtered. Intensity was defined as ≥50 eggs per 10 mls of urine. Please see line 261 and 266-268.*

Genetic profiling in general – as your data is based on pooled samples you cannot precisely say what the genetic profiled is for all your samples. You can say you have signal from the different species in the different markers. It is surprising that you did not get cox1 and ND5 sequences from both Sb and Sh in your individual samples?

*We appreciate the reviewer’s perspective, what was surprising to us, was that we only ever got one homozygous sequence type, with no heterozygosity detected at the PCR population level. In the majority of our samples, at least two independent PCR reactions were amplified and sequenced (both directions), with the same results found, which rather supports a hybrid origin, than infections with a multitude (or mixture) of parasite species that are discordant at their mitochondrial genome. Hence, we did not get any signal for both Sb and Sh in the individual samples analysed.*

Line 137 – this SNP has been identified before – reference Savassi et al., 2020.

*We thank the reviewer; we have added this reference to the paper. Please see line 145 in the revised manuscript.*

Line 149 – when you say “allele” – do you mean haplotype?

*We thank the reviewer for bringing this to our attention, Yes, we mean haplotype.*

In the text it would help if you referred to specific samples in the table when you are discussing the paper – a good example of this is in line 154.

*We thank the reviewer for bringing this to our attention, sample IDs have been included in the text for the most relevant associations found for samples OD14, OD17 and J15, for example.*

It is surprising when talking about the haplotype diversity for S. haematobium you do not reference Webster et al., 2012.

*We thank the reviewer for bringing this to our attention, the reference has been included in the text. See line 147, Reference No. 16*

Lines 168-174 – much of this is methods and data should be shown for the testing of the markers on the different species. Same for the ND5.

*We thank the reviewer for their perspective, the amplification of both* BF *and* ND5 *was robust for all species tested, so we do not feel that it is necessary to add the data to the paper. However, we do agree that the sequences are important, so we have added the species-specific sequences into GenBank, please refer to accession IDs OR574883-OR574888 for BF and OQ571658-OQ571713 for ND5. We also added these Accession IDs into the respective phylogenetic tree figure legends and tree for the different species accordingly: (Fig 4; BF) and (Suppl. Fig 2; ND5).*

For the BF markers – you need to explain if this signal is based on SNP’s or a sequence – as it is a protein coding gene it would be expected to be based on a sequence and so it is not clear how you analysed the data when it was heterozygous. Would you not expect natural variation in the BF target?

*We thank the reviewer for bringing this to our attention, as we indicate in our paper, across the 408 nucleotide amplicon sequence, 29, 14, 9, and 3 SNPs separate the S. haematobium BF allele from that of S. mansoni, S. bovis, S. curassoni and S. mattheei reference strains (please see lines 182-186). To develop the primers, we downloaded sequences from NCBI for the BF marker across all species of schistosomes thus far studied, performed an alignment in Geneious, and identified only those regions that had high sequence conservation across all species but bracketed polymorphic stretches to identify the 408 nt sequence used in this study. These details have been added to the Materials and Methods section in our revised manuscript, please see lines 309-317.*

Line 195 – the nested configuration was not clear in the methods

*This has been added to the methods, please see lines 309-310*

The small conclusion is a good overview of the complexed nature of S. haematobium and these hybrid forms and notes the key future steps needed to fully understand the epidemiology of these species complexes. I think the paper would benefit from a distinct discussion section

*We thank the reviewer for their perspective, as we indicated earlier, we believe it best to combine and we trust this will be okay with the reviewer.*

The paper could present important data. However, there are many details missing and the results are not presented clearly. The text should be read carefully to correct mistakes and also to improve clarity.

*We hope this has been addressed to the reviewer’s satisfaction in our revised submission.*

The paper presents molecular data from pooled Schistosoma egg samples isolated from individual children in Nigeria. The authors have used traditional markers for hybrid detection (ITS and cox1) and have also used two additional markers ND5 and Bravo-Figey region to support the data interpretation. The authors present novel data that is informative however, more detail on how the data was generated and analysed is needed before the paper can be reviewed further.

*We thank the reviewer for their positive comments, and in our revised submission, we have integrated the 4 markers to provide what we believe is the perspective the reviewer is requesting.*

The genetic profiles presented from the eggs are interesting and complexed and the results need to be presented in a clear way to allow interpretation. By using other papers that have presented similar datasets as a guide, the authors will be able present the data clearer.

*We thank the reviewer for this perspective, we decided to re-draw the distance-based trees, to show the sequence type for each sample, to better demonstrate the total number of alleles present and their frequency at each locus.*

**Reviewer #2:**

Minor and some more major revisions have been detailed below. The study artciulates testable hp and objectives, but details about the study design are missing (and discrepancy between the age of the population surveyed is present throughout the text). Concerns about statistical approaches and other clarifications to ensure compliance to ethical requirements have been highlighted in the comments below.

*We thank the reviewer for pointing this out, we have addressed this in our comments to reviewer #1 above.*

The results are clearly presented but modifications and further details are necessary. These have been highlighted in the comments below.

The public health relevance and the conclusions of the study are effectively addressed.

*We thank the reviewer for their positive comment.*

LINES 32-33. The first time a scientific species name is mentioned it should be fully spelled out. The same goes for the genomic markers (and refer to them as ITS and cox1 rather than transITS and CO1). Modify here and throughout the text.

*We thank the reviewer for bringing this to our attention, we have provided our rationale for using the transITS under a similar comment by Reviewer 1. We also understand that CO1, which is synonymous with cox1, is the preferred acronym for the locus as part of the international Tree of Life and Barcoding initiatives, to which we subscribe. We trust the reviewer is fine with us continuing to refer to these loci as trans*ITS *and* CO1. *Of course, if the reviewer objects, we would be happy to change in any future revision of the manuscript.*

LINE 38.  Use Sh x Sb abbreviated since you already specified it above.

*We thank the reviewer for bringing this to our attention, the text has corrected in the revised manuscript.*

LINE 53.  May pose rather than poses.

*Corrected.*

LINES 80-83. This paragraph may be missing a reference, the provided [4] seem to refer to work done in Senegal.

*We thank the reviewer for bringing this to our attention, the correct reference (see reference [3]) has been placed in the revised manuscript.*

LINE 93. More than failure, those studies highlighted the absence of ongoing hybridization events.

*We thank the reviewer for bringing this to our attention, the text has corrected in the revised manuscript, please see below:*

*“The absence of full genome hybrids was interpreted to suggest that interspecies…”*

LINES 119-122.  Refer to the specific site names to avoid confusion.

*We thank the reviewer for bringing this to our attention, the text has been corrected in the revised manuscript, please see below:*

*“In* *Ile Oluji/Oke Igbo that was devoid of livestock, 17 of 107 filters contained parasite eggs. DNA was extracted from these S. haematobium positive urine samples. Forty of 112 filters contained parasite eggs in the pastoral community in* *Kachi, and DNA was extracted from these S. haematobium positive urine samples.*

LINE 134.  Modify to a Sanger sequencing strategy.

*We thank the reviewer for bringing this to our attention, the text has corrected in the revised manuscript, please see below:*

“*…a Sanger sequencing strategy…*”

LINE 160.  Table 2 is missing a legend.

*We thank the reviewer for bringing this to our attention, in our revised submission, we have included a Table 2 title, and legend. Please see Table 2, line 171, 172-176*

LINES 204-205.  I would remove this sentence since this study does not really demonstrate ITS and cox1 being inadequate.

*We agree. In our revised manuscript, we modified this sentence accordingly, please see lines 214-217.*

*“Although the CO1 and transITS gene markers are reasonably proficient at detecting hybrids, the inclusion of additional markers allows for a better resolution of complex interspecies hybridization events, including livestock only hybrids, as evidenced herein that was consistent with a cross between an Sh strain with an Sc x Sb livestock:livestock hybrid that infected two individuals.”*

LINE 217.  The authors did a great job to stay away from the word “pure” until the very end. Rather than using this, clarify the need for WGS of multiple S. haematobium coming from areas where livestock schistosomes are absent.

*We thank the reviewer for bringing this to our attention, we have made changes in the revised manuscript. Please see line 229-230.*

*“Finally, there is a need for whole genome sequencing analysis of multiple S. haematobium samples from areas where livestock schistosomes are absent.”*

LINE 225.  Specify the institution providing ethical clearance and why purely oral consent was sought and what has been done to demonstrate that such consent was obtained.

*We thank the reviewer for bringing this to our attention. In our revised manuscript, we have added this information, please refer to lines 239-240*

*“The study was conducted in rural communities where the villagers provided only oral, not written, consent.”*

LINES 246-248. Specify the technique for the microscopical visualisation of the eggs.

*We thank the reviewer for bringing this to our attention. We have addressed similar comments by Reviewer 1 in our revised manuscript, please refer to line 263-268.*

LINES 254-256. Where the positive controls worms or DNA samples? Please acknowledge again the source (as you did in the Acknowledgement session). Spelling mistake for QIAGEN.

*We thank the reviewer for bringing this to our attention. We have addressed similar comments by Reviewer 1 in our revised manuscript, please refer to line 278-281. We also have added the sources for the worms in this Section, in addition to the Acknowledgements section.*

LINES 263-264.  Specify that also these S. bovis-specific primers were used to amplify nad5.

*This has been removed in our revised submission, per Reviewer #1.*

LINE 274.   Which institution Sanger sequenced the samples and what machine was used?

*All Sanger sequencing was performed at the Rocky Mountain Laboratory, National Institutes of Health, in Hamilton, Montana USA.*

LINES 282-284.  When looking at the figures, I realised that each gene was modelled by itself; therefore, it would be good to clarify that a different model was implemented for each marker. Also, why did you decide not to concatenate mitochondrial gene sequences and preferred to analyse them separately (same goes for nuclear ones although heterozygosity may have complicated this)?

*We thank the reviewer for their perspective, we did indicate that each tree was for a single marker. We chose not to concatenate, largely due to the high degree of hybridization at the nuclear markers. But equally, it was our preference to be transparent with the mitochondrial data, to show that very little genetic drift had occurred among our samples.*

LINES 330-335.  Revise references 7 and 8; the remaining literature seems well written.

*We thank the reviewer for bringing this to our attention, the text has been corrected in the revised manuscript.*

General remarks:

This would be an interesting manuscript given the sampling efforts and the ability to connect field and laboratory data within the wider epidemiological context of schistosomiasis in Nigeria. The findings are relevant and add a piece to the puzzle of Schistosoma hybridizations across sub-Saharan Africa. I have highlighted some areas where the authors need to modify the manuscript, or at least provide some further details.

Major comments.

LINES 261-271.  What software was used to design those primers? Why only PCR conditions for 2 genes were reported? This might be my ignorance, but I personally never came across Bravo Figey, is this the first study using this nuclear marker in Schistosoma or, more in general, helminths? If not, provide a brief rationale that drove the selection of this marker and conditions for the nested PCR.

*We thank the reviewer for bringing this to our attention, we addressed this in our response to Reviewer 1, who had a similar query. Please see lines 308-319.*

LINES 123-127.  The type of statistical tests that have been executed are not reported in the Methods. I assume these may be all binary t-tests, what justifies their application instead of a generalised model considering all the epidemiological features? Lastly, age may be significant but profiles between the 2 sites seem to tell rather different stories, how would you explain it?

*We thank the reviewer for bringing this to our attention. In our revised manuscript, we have added this information, please refer to lines 268-269.*

*“Statistical analysis was done using the Fisher exact test to compare the prevalence and intensity of infection with demographic variables.”*

DISCUSSION.  The details about the different haplotypes are great but the Results-Discussion do not seem to convey a relevant finding of this study: the semi-complete split between the schistosomes circulating across Jigawa against those across Ondo.

*We thank the reviewer for bringing this to our attention. In our revised submission, we discuss the probable origin of the zoonotic livestock-specific schistosomes that were identified, and that the current two genetic markers applied were insufficiently resolved to capture the hybrid livestock:human schistosomes, nor were they able to discriminate the event that was responsible for their origin. We also added this perspective in our revised conclusion.*

FIGURE 4. At those sites of heterozygosity, how did the authors decide how to code their base call? The same goes for Supplementary Figure 1 about the ITS marker. I feel that these 2 figures, and their text, could be removed unless clear explanation is provided about base calling, which sequences exactly the authors used for phylogenetic inference, and what information these trees add that is not already provided in Table 2.

*We thank the reviewer for their perspective. As we indicate in our above response to Reviewer #1, at least two independent PCR reactions were amplified and sequenced (in both directions) for every sample, with the same results found, which, combined with the identification of only a single homozygous sequence type at both mitochondrial markers utilized, the data rather supports a hybrid origin, than infection with a multitude (or mixture) of parasite species that are discordant at their mitochondrial genome. Once this was established, the most parsimonious explanation for the origin of the heterozygosity observed at the transITS marker was that of the existence of two haplotypes that reflected the hybrid origin of the sample. Our sequencing at transITS of the typed specimens from the various species (S. mansoni, S. curassoni, S. bovis, S. mattheei*, and *S. haematobium) only identified a single homozygous sequence type. For each sample that possessed sites of heterozygosity, it was possible to phase the haplotype based on the reference sequences, as this was the most parsimonious explanation for the origin of the heterozygosity, given the single, homozygous sequence type identified in each sample at the 2 mitochondrial markers. Hence, we feel it is informative to include this analysis in the paper, and that the base calling/phasing is justified. We have included this justification in our revised manuscript in the Molecular analysis and Phylogenetic analysis sections within the Materials and Methods.*

**Reviewer #3:**

Overall, the authors' methodology is sufficient to address the questions posed and consistent with similar studies/publications in the field.  There are just a few minor points/suggestions I would like the authors to consider:

1 - The authors describe the agrarian site as being "devoid of livestock", but I think the support of their chosen sites would be improved if this description was made more epidemiologically clear.  Not to be glib, but how devoid is "devoid"? At what geographical scale is the study area "devoid" of livestock relative to the average regular movement and water usage patterns of the local human populace?  Is there literally no opportunity for shared human-livestock water source usage outside of a rare long-distance trip by a resident?  No opportunity for rodent hosts to occupy a geographical hybrid zone between the agrarian area and a neighboring area where livestock ownership is more prevalent?

*We thank the reviewer for bringing this to our attention. The selected communities are established field sites that have been monitored over a few years. However, we do agree with the perspectives of the reviewer on human migration and parasite transmission. In our revised submission we clarify the term “devoid of livestock” to refer to an absence of human-cattle water contact activities. In these field sites, cattle rearing and/or cattle markets are absent within the community. Please see lines 121-123. We did not account for rodent hosts under this description as there was no previous evidence of zoonotic schistosome species in the area.*

1b - The way that the authors refer to the study sites would benefit from some more consistent language.  At times the sites are referred to by their name and at others by the methodological descriptors (e.g., "pastoral" vs. "agrarian"/"devoid of livestock").  For the sake of clarity and reader efficiency, I would suggest picking a reference and being consistent in its use -- ideally something that relates directly to the study question/methodology.

*We thank the reviewer for bringing this to our attention. We have selected agrarian and pastoral* for *the description of sites to avoid confusion. The definition of agrarian is included at the first mention in the revised manuscript. Please see lines 105-107.*

*“The sampled community in southern Nigeria is agrarian in nature, devoid of livestock i.e., agricultural activities are predominant and cattle rearing and/or markets are absent whereas the community in the northern part of the country is pastoral.”*

2 - As currently presented in the methods section, total DNA used for the sequencing was derived from pools of eggs (Line254: "Total DNA was extracted from individual filters..." -- not from individual eggs or miracidia).  That is acceptable methodology, but the term "isolate" is consistently used throughout the manuscript to report and discuss results in a manner that could lead a reader to the conclusion that the results deal with individual larval genotypes, rather than profiles of allele presence/absence in a pooled sample (e.g., "isolate J15 was heterozygous" - Line176).

*We thank the reviewer for bringing this to our attention, we addressed this in our response to Reviewer 1, who had a similar query. The term isolates have been replaced with samples in the revised manuscript.*

Data in Table 2 are presented in such a way that imply a single isolate's genotype or that all individuals in the sampled pool have the same patterns of zygosity.  For instance, it is clear that the results for Ondo are consistent with widespread ("universal"?) mitonuclear discordance, but are the authors suggesting that all of the eggs in the pool comprising sample OD1, for example, are heterozygous for S.h. and S.c. alleles at the ITS locus?  It may just be a matter of degrees, but that would have a different epidemiological implication than finding that a subset of individual larvae are the product of S.h.xS.c.xS.b. hybridization.  If the authors are suggesting that all of the larvae in the OD1 pool are, in fact, S.h.xS.c.xS.b. hybrids, then there should be some inclusion of the assessment of the relative peak height of heterozygous sites in the methodology and/or results that support that conclusion.  This is especially true for the results from Kachi, where one could envision the potential for variability of hybrid prevalence.  Using relative peak heights to derive an estimate of hybrid prevalence in Kachi could be an interesting addition.  But, more importantly, I would urge more clarity/transparency in the presentation of the results.  At a minimum, remind the reader early on in the Results section that the data for "isolates" are derived from pools of samples and reference the limitation in the ability to provide an absolute prevalence of the various hybrid combinations.

*We completely agree with the perspective raised by the reviewer, and this point was specifically echoed by both reviewers #1 and #2. In our revised submission, we carefully point out that at least two independent PCR reactions were amplified and sequenced (in both directions) for every sample, with the same heterozygous positions found in all repeat samples, which, combined with the identification of only a single homozygous sequence type at both mitochondrial markers utilized, the data rather supports a hybrid origin, than infection with a multitude (or mixture) of parasite species. Hence, at the PCR population level, we were capable of resolving the sites of heterozygosity with high confidence, in both FWD and REV sequence reads, and independent replicates, and in all cases, the positions that were heterozygous, remained heterozygous, and could be resolved. We do not want to reference peak heights as this is too subjective, and it would be better to develop an NGS single molecule sequencing pipeline to infer relative prevalence. In addition, we think it is important, in a follow-up study, to sequence hatched miracidia to confirm the widespread, or universal occurrence of the patterns of zygosity observed.*

3 - For several sample pools, analysis of CO1 was reported as "not done" (Table 2).   Please clarify whether these were failed PCR/sequencing reactions or whether there was a conscious decision not to collect CO1 data for those specimen and, if so, why not.

*We thank the reviewer for bringing this to our attention, the term – n.d. – “not defined” has been clarified in Table 2 as “failed PCR/sequencing reaction” per reviewer’s suggestion in the revised manuscript.*

4 - Very minor: in the Materials and Methods please report the source of the positive control materials.

*We thank the reviewer for bringing this to our attention, the section has been revised to address similar comments raised by the Reviewer 1. See lines 278-281 in the revised manuscript.*

Table 1 -- This table is titled as "S. haematobium prevalence..." but prevalence is only 1 of 4 metrics presented and the title could lead to confusion as it stands (or at least require the reader to spend more time deciphering the information).  It would be more precise to say that it is "Study population demographics and S. haematobium prevalence", or something comparable.

*We thank the reviewer for bringing this to our attention. In our revised manuscript, we have added this information, please refer to lines 132-133 accordingly:*

*“Table 1: Schistosoma haematobium prevalence and study population demographics in Ile Oluji/Oke Igbo (Ondo State) and Kachi (Jigawa State), Nigeria.”*

Figure 3 -- The color differences should be more apparent and should be tested for color blind friendliness (e.g., via the Coblis [<https://gcc02.safelinks.protection.outlook.com/?url=https%3A%2F%2Fwww.color-blindness.com%2Fcoblis-color-blindness-simulator%2F&data=05%7C01%7Cgriggm%40niaid.nih.gov%7C6c45c48cf7c64931d8b408dba59a2359%7C14b77578977342d58507251ca2dc2b06%7C0%7C0%7C638285853688542068%7CUnknown%7CTWFpbGZsb3d8eyJWIjoiMC4wLjAwMDAiLCJQIjoiV2luMzIiLCJBTiI6Ik1haWwiLCJXVCI6Mn0%3D%7C3000%7C%7C%7C&sdata=sLpRZB6FCL9kloaIarR4fH0ZE7X2UhhZFLZIZmpD95M%3D&reserved=0>] or Color Oracle [<https://gcc02.safelinks.protection.outlook.com/?url=https%3A%2F%2Fwww.colororacle.org%2F&data=05%7C01%7Cgriggm%40niaid.nih.gov%7C6c45c48cf7c64931d8b408dba59a2359%7C14b77578977342d58507251ca2dc2b06%7C0%7C0%7C638285853688542068%7CUnknown%7CTWFpbGZsb3d8eyJWIjoiMC4wLjAwMDAiLCJQIjoiV2luMzIiLCJBTiI6Ik1haWwiLCJXVCI6Mn0%3D%7C3000%7C%7C%7C&sdata=5MoyJVBZ2EAZTbFOnKI1iInsR4jgs2Mzfj86rAfgZG8%3D&reserved=0>] tools).  The blues and sea green are particularly hard to distinguish.  Also, if possible, please work with the angles of the small S. bovis branches to further separate and clarify any forks and assist with resolving the individual circles.

*We thank the reviewer for this comment. In our revised manuscript, we have improved the figure and use an appropriate color scheme according to the links above. Please see figure 3.*

Overall, the conclusions seem to correspond to the results and methodology, though a key alternative explanation to the observed hybrids in the agrarian zone is not presented -- that of ancient introgression instead of active hybridization. The inability to distinguish ancestral introgression from contemporary hybridization with the methodology they are using should be noted.  They are assuming that their results indicate contemporary hybridization, while many of the genomic studies they cite only report evidence for ancestral introgression.  That limitation should be included as a caveat to the interpretation of the results, and they might also find it helpful to include the recent paper by Berger et al. (Genomic evidence of contemporary hybridization between Schistosoma species, 2022, PLoS Pathog) to lend support to the notion that the results presented here could feasibly be due to contemporary hybridization.

*We thank the reviewer for their perspective, we whole-heartedly agree. In our revised submission, please see lines 163-169, we added the two explanations highlighted above, and we included the recent paper by Berger et al. 2022 that supports our prevailing tenet, that hybridization is an ongoing process in the origin of new zoonotic strains with an expanded host range, disease potential, and tropism. In no way does this perspective exclude the existence of ancient introgression, but rather that hybridization is a natural process in the transmission of this highly successful species complex.*

For the most part, this manuscript is clearly communicated.  The authors have sequenced 4 loci (2 nuclear and 2 mitochondrial) in pools of S. haematobium eggs to investigate the presence of human-animal schistosome hybrids in two regions of Nigeria that differ in the prevalence of livestock and, thus, theoretically the risk of parasite hybridization.  Their work would add to the collection of studies purporting to show evidence of hybridization, though it should be noted that the role of ancestral versus contemporary events in producing these observations is still not particularly clear.  I recommend acceptance with minor revisions to enhance clarity and transparency.

*We thank the reviewer for their positive comments and support.*
