# Supplementary material for: Extant interspecific hybridization among trematodes within the Schistosoma haematobium species complex in Nigeria
Source: PLoS Negl Trop Dis. 2024 Apr 15;18(4):e0011472. doi: 10.1371/journal.pntd.0011472 (PMC11045100; doi:10.1371/journal.pntd.0011472)
Supplement: Attachment — Submitted filename: 24 PNTD-Response to Reviwers_MEG_020524.docx [file pntd.0011472.s006.docx]

"Extant interspecific hybridization among trematodes within the *Schistosoma haematobium* species complex in Nigeria"

*We thank the editor for their positive comments, we have revised our manuscript accordingly and trust that it is now acceptable for publication. We believe our manuscript is much improved. Please find our responses to the editor’s comments (in blue), a “clean” and “track-changes” copy of the revised paper.*

The most important thing is to include some caveats around the interpretation of "hybrids" from pools of eggs, and I’m not sure the response to the reviewer #1 and #3 comments is really completely adequate. In particular, finding identical results in two independent PCR reactions may not be so surprising if analysis is from a large pool of individual miracidia.

*We thank the editor for bringing this to our attention, we agree. the discussion section has been revised to address your concerns. However, we think it more prudent to flip the discussion, as we did not expect to see persistent, reproducible, equal peak heights for the bi-allelic SNPs at sites of heterozygosity, which repeated with high confidence between independent replicates, and rather supported a strictly “hybrid” interpretation. Please refer to the eletropherograms provided from sequencing pools of eggs from different patient samples in Supplemental Table 2. For example, in sample OD17, only one single homozygous sequence type was resolved across independent PCR reactions, that possessed an Sc allele (the polymorphism at position 19 was an “A” which differentiates Sc from Sb, the latter possessing a “G” nucleotide). Further, the electropherograms from two other patients (OD9, OD12) that represent the majority of samples, had similar results, and in all cases the positions that were heterozygous, remained heterozygous, with equal peak heights, which is parsimonius with a hybrid origin, rather than infection with a genetically distinct mixture of different parasite species, in which case the peak height and heterozygosity would be more variable . Yes, our samples were pools of eggs, and we expected, given the prevailing “genetic mixing bowl” hypothesis that presupposes greater parasite diversity in endemic zones due to continuous re-infection of an already infected host pool [6], that this would lead to an increased number of paired schistosomes, and that peak height would be quite variable after sequencing the PCR population, and reflect a heterogeneity of different schistosome genotypes that are present within an animal host. This was not the case. To further address this perspective, we would need to prospectively collect new samples from the same geographic sites, and perform PCR on both pools of eggs, as well as the individual miracidia hatched from these collected eggs to infer more precisely the number of distinct haplotypes present in the parent population that lives within the sampled human patients. This is beyond the scope of our current publication. In fact, our expectation was that, as has been seen, for example, when individual miracidia were hatched from pools of eggs collected from patients in Niger [17], that a diverse number of haplotypes would be recovered within the miracidia, and that this would have led to highly variable peak heights at sites of heterozygosity after sequencing the PCR population, which would not necessarily replicate between independent PCR reactions. Which I think is the basis for the editor’s comments above. We hope we have addressed this concern in our resubmission. Please see lines 159-170 in our revised resubmission.*

I think this also relates to the concerns reviewer #1 has about a lack of discussion section: as at the very least something needs to be said to make sure it is abundantly clear that the restults are (strictly speaking) findings of diversity of markers in pools of miracidia, rather than heterozygosity of individual organisms. I would like to see several sentences explaining this clearly and explaining the haplotype inference so readers who don't delve into the methods are not misled.

*We thank the editor for bringing this to our attention, we have added comments within the text, that the results were derived from sequencing pools of eggs, and more to the point, that we did not find a diversity of sequence types, which would be the expectation from sequencing a pool of eggs containing a diversity of different genotypes of miracidia. Rather, we only ever got a highly reproducible sequence type that supported limited genetic diversity, and a strictly “hybrid” interpretation. But we also take the editor’s point, so we are more circumspect in our revised resubmission, and we now use the phrase “genetic profiles” rather than “haplotypes” in the majority of instances to reflect the fact that we have sequenced from pools of eggs, but that the genetic profile identified was uniform (and we included examples of our DNA sequencing electropherograms to demonstrate this point – see Supplemental Table 2, panel B), and that the most parsimonious explanation for our results is that of limited diversity, akin to a hybrid population from a limited number of worm pairings in infected hosts.*

it would also be useful to see some description of what the authors think is going on in these hybrid populations. As schistosomes are obligately sexual in the adult stage, we might expect to see something like Hardy-Weinberg proportions of heterozygous and homozygous markers, but instead these populations appear to be extremely heterozygous. How do they consider this is likely to come about? To my mind, the obvious explanation would be that the F1 hybrids are infertile, but that doesn't tie in with the proposed 3-way hybridisation. I'd be interested to see the author's proposal for the possible biology here, and think that would make some useful discussion.

*We thank the editor for asking this question. It is our view that no mating incapabtibility likely exists for worms within the S. haematobium species complex. We believe that prospective studies need to be carried out using an increased number of genetic markers, or at whole genome resolution, on miracidia isolated from patient egg samples that appear to have interspecific infections to truly determine the extent of mixed infections, and the haplotypes that can be generated from cross-pairing among the various species within the S. haematobium species complex. These comments have been made in our revised resubmission, please see lines 201-212.*

Some minor comments:

*We thank the editor for bringing these minor comments to our attention. Each has been addressed accordingly, as listed below.*

line 38: could be made clearer that there were only 57 positive samples.

Changed to: “Out of a total of 219 urine samples collected, 57 were positive for schistosomes.” Line 37 in our revised manuscript

line 77: "It is the second most prevalent tropical disease caused by five major species" - this needs re-wording, as it currently implies that a more prevalent disease is caused by the same 5 species. It needs 'and is caused by'.

Changed, thanks. We also failed to include *S. guineensis, so we have added that in our revised manuscript.* Please see lines 69-70.

line 89: There is a formatting error here: [6, 7,] (delete final comma)

Done.

line 93: 'resulting in only' would be better as 'at only'

Done.

line 121-123. – It might be good to say something about how far away the nearest cattle-rearing area is? That might speak to the possibility of transmission mediated through rodents etc. etc.

We thank the editor for their perspective, but we actually do not have any current information on the nearest cattle-rearing area. Nomadic farmers are constantly on the move in search of pastures in Nigeria.

line 143: Of particular interest, a private SNP at nucleotide 502 (based on GenBank reference sequence MH014047) was identified that was unique to strains circulating in Kachi, this has been previously observed by Savassi et al, (2020) [15] in S. haematobium or S. bovis samples obtained from human infections (Suppl.Table 2).

IT would be useful for readers (and editors) to explicitly point out which SNP in the Savassi et al. paper position 502 here represents, as they seem to be on different co-ordinate systems. I guess this is 556 in the Savassi et al?

We thank the editor for this comment, we have changed the text accordingly, please see lines 136-137 in our revised resubmission:

*“Of particular interest, a private SNP at nucleotide 502 (based on GenBank reference sequence MH014047) was identified that was unique to strains circulating in Kachi, this SNP has been identified previously at position 556 in the co-ordinate system used by these investigators [15] among S. haematobium or S. bovis samples collected from human infections (Suppl.Table 2).”*

line 145: by Savassi et al, (2020) [15] - it would be better 'style' to just say [15]

Done.

line 154: 'that resolved from other human and livestock schistosomes' - doesn't read as quite grammatical to me. I think this needs some extra words? - maybe something like 'resolved as a monophyletic group excluding other human..'

We thank the editor for their comment, in our revised manuscript, we changed the text accordingly: “…from the public domain, that resolved as a monophyletic group and was distinguished from other human and livestock schistosomes within the *S. haematobium* species complex…” please see lines 148-149.

line 217 : hyrid -> hybrid

Done.

line 226: individually genotype miracidia -> I think 'genotype individual miracidia' would be clearer here.

Done.

line 264 and 265: S. haematobium should be in italics.

Done.

line 268- 269: Statistical analysis was done using the Fisher exact test to compare the prevalence and intensity of infection with demographic variables.” -- Does this make sense? are all such variable categorical?

Yes, all variables are categorical, please see Table 1.

line 279:

'and an S. bovis worm (extracted from a cow in Nigeria)'

How confident can the authors be that this is a "pure" S. bovis? Does it matter?

We thank the editor for their perspective, we agree. It was included as a local reference, and at four markers, it types as *S. bovis* – but whether it is *S. bovis* genome-wide is another matter. It does not matter.

in your response to review #2, you refer to the postiive controls as "typed specimens", and while I guess the SCAN specimens were typed by the collectors, you don't say anything about how this was 'typed'.. or is assumed that cattle schistosomes in that area are 'pure' S. bovis.?? Something should be said.

It was typed at the four markers used in our study, as stated above.

line 280: 'Pure Genomic DNA' - not sure what this means, so would be good to have some explanation?

We thank the editor for pointing this out. We agree, and in our revised submission, we dropped the word “pure” and start the sentence with “Genomic DNA was obtained…” Please see line 291.

Lines 316-317 - I think it would be good to at least add a statement about the degree of testing. How many samples were tested of each species? If more than one, was amplification (and sequence data) identical in every case?

Done, please see lines 333-335 in our revised resubmission.

The writing goes a bit wrong at line 337:

"was implemented using MEGA X based on the best model (lowest Bayesian information criterion score) for each gene marker [29], using Maximum Likelihood method at 1000 bootstrap replicates to ascertain the reliability of the tree branches. Haplotype network was implemented for CO1 sequence data in an R environment using the Pegas PE package [30]."

We thank the editor for pointing this out, we have revised the text accordingly, please see lines 347-350 in our revised resubmission.
