# Supplementary material for: Extant interspecific hybridization among trematodes within the Schistosoma haematobium species complex in Nigeria
Source: PLoS Negl Trop Dis. 2024 Apr 15;18(4):e0011472. doi: 10.1371/journal.pntd.0011472 (PMC11045100; doi:10.1371/journal.pntd.0011472)
Supplement: Attachment — Submitted filename: 24 PNTD-Response to Editor_MEG_032124.docx [file pntd.0011472.s007.docx]

"Extant interspecific hybridization among trematodes within the *Schistosoma haematobium* species complex in Nigeria"

*We thank the Section Editor for his positive comments, we have revised our manuscript accordingly and trust that it is now acceptable for publication. Please find our responses to the editor’s comments (in blue), a “clean” and “track-changes” copy of the revised paper.*

I would only like to point out two aspects of the manuscript that may have escaped the previous rounds of review:
- Regarding Table 1: if I understand correctly, the p-values refer to tests that are comparing the two locations (Ile Oluji/Oke Igbo vs Kachi). e.g. both locations are significantly different regarding their total prevalence, but not their infection intensity. In the case of age, it would thus seem that the p-value of < 0.00001 refers to the different age composition of the samples at both locations. However, the main text stages that "Age was significantly associated with infection (p<0.00001)" (l. 132) which does not seem to have been tested. It also says "...as has been reported previously" but does not provide a reference.

*We thank the editor for bringing this to our attention, you are correct. The p-value does indeed refer to a significant difference in the age composition between the two locations. In our revised maunsript, we have changed the text accordingly, to reflect that point:*

Line 119-121: The age of the participants ranged from 3–19 years and differed significantly between the two locations (p<0.00001). The prevalence was higher in males (55.8%) than females (45.2%), but this did not reach significance (Table 1). In both study sites, a high intensity of infection was recorded (≥50 eggs per 10 ml of urine) (Table 1).

- The legend to Table 1 says "Prevalence percentages are in parenthesis" - however, this is not the case, the numbers in parentheses are only showing the % for each category of each variable.

*We thank the editor for bringing this to our attention, we have changed Table 1 legend to reflect that “percentages are in parentheses”*

- Line 215 "The presence of such hybrid Sb x Sc livestock:human Sh genotypes have been described previously  and support a model whereby no real or perceived barrier of genetic hybridization exists between livestock and human species of trematodes within the S. haematobium species complex. " - a reference appears to be missing here.

*We thank the editor for bringing this to our attention, the missing reference is [17]. This has been added in our revised resubmission, please see line 203.*
